# Impact of Chromosome Fusions on 3D Genome Organization and Gene Expression in Budding Yeast

**DOI:** 10.1534/genetics.119.302978

**Published:** 2020-01-06

**Authors:** Marco Di Stefano, Francesca Di Giovanni, Vasilisa Pozharskaia, Mercè Gomar-Alba, Davide Baù, Lucas B. Carey, Marc A. Marti-Renom, Manuel Mendoza

**Affiliations:** * CNAG-CRG, The Barcelona Institute of Science and Technology (BIST), 08028 Barcelona, Spain; † Centre for Genomic Regulation (CRG), The Barcelona Institute of Science and Technology (BIST), 08003 Barcelona, Spain; ‡ Institut de Génétique et de Biologie Moléculaire et Cellulaire, 67404 Illkirch, France; § Centre National de la Recherche Scientifique, UMR7104, 67404 Illkirch, France; ** Institut National de la Santé et de la Recherche Médicale, U964, 67404 Illkirch, France; †† Université de Strasbourg, 67000 Strasbourg, France; ‡‡ Center for Quantitative Biology, Academy for Advanced Interdisciplinary Studies, Peking University, 100871 Beijing, China; §§ Peking-Tsinghua Center for the Life Sciences, Academy for Advanced Interdisciplinary Studies, Peking University, 100871 Beijing, China,; *** ICREA, 08010 Barcelona, Spain; ††† Universitat Pompeu Fabra (UPF), 08002 Barcelona, Spain

**Keywords:** budding yeast, computational modeling, gene expression, nuclear organization, single-cell imaging

## Abstract

The three-dimensional (3D) organization of chromosomes can influence transcription. However, the frequency and magnitude of these effects remain debated. To determine how changes in chromosome positioning affect transcription across thousands of genes with minimal perturbation, we characterized nuclear organization and global gene expression in budding yeast containing chromosome fusions. We used computational modeling and single-cell imaging to determine chromosome positions, and integrated these data with genome-wide transcriptional profiles from RNA sequencing. We find that chromosome fusions dramatically alter 3D nuclear organization without leading to strong genome-wide changes in transcription. However, we observe a mild but significant and reproducible increase in the expression of genes displaced away from the periphery. The increase in transcription is inversely proportional to the propensity of a given locus to be at the nuclear periphery; for example, a 10% decrease in the propensity of a gene to reside at the nuclear envelope is accompanied by a 10% increase in gene expression. Modeling suggests that this is due to both deletion of telomeres and to displacement of genes relative to the nuclear periphery. These data suggest that basal transcriptional activity is sensitive to radial changes in gene position, and provide insight into the functional relevance of budding yeast chromosome-level 3D organization in gene expression.

## Abstract (precis)

In eukaryotic cells, the spatial organization of genes within the nucleus is correlated with their expression. However, correlation is not causa-tion. To determine how nuclear spatial organization affects gene expression, Di Stefano et al. studied...

CHROMOSOMES in interphase nuclei are spatially distributed in a nonrandom manner. Indeed, chromosomes are organized in distinct structural units and their organization influences nuclear functions such as transcription, replication, and DNA damage repair [reviewed in Gibcus and Dekker (2013), Furlan-Magaril *et al.* (2015), Lemaître and Bickmore (2015), and Denker and De Laat (2016)]. In animal cells, individual chromosomes tend to occupy defined nuclear regions termed “chromosome territories” (CTs) (Cremer *et al.* 1982; Haaf and Schmid 1991; Cremer and Cremer 2001; Branco and Pombo 2006), and the spatial distribution of CTs can be size- and gene density-dependent. In several cell types, gene-poor chromosomes associate preferentially with the nuclear periphery, whereas gene-rich chromosomes are enriched in the nuclear interior (Croft *et al.* 1999; Boyle *et al.* 2001). In addition, distinct structural domains at the subchromosomal level have been identified by microscopy, termed chromosomal domains (Markaki *et al.* 2010). Chromosomal domains may correspond to subchromosomal units defined by their increased interaction frequencies with each other or with the nuclear lamina. In particular, the nuclear periphery is a transcriptionally repressive environment in yeast and metazoans (Andrulis *et al.* 1998; Pickersgill *et al.* 2006; Guelen *et al.* 2008; Green *et al.* 2012), and gene repositioning from the nuclear interior to the periphery leads to repression of some, but not all, genes tested (Kosak *et al.* 2002; Zink *et al.* 2004; Kumaran and Spector 2008; Reddy *et al.* 2008; Finlan *et al.* 2008). Notably, individual genes can display mobility within chromosomal and subchromosomal domains, and this has been correlated with changes in their expression levels during cell differentiation (Peric-Hupkes *et al.* 2010). However, it remains unclear if the position of individual genes within the nucleus affects their expression, and/or their ability to be silenced or activated in response to different stimuli, or if these expression-related properties are merely correlated with spatial organization.

Studies in the budding yeast *Saccharomyces cerevisiae* have provided insight into the functional role of nuclear spatial organization [reviewed in Taddei *et al.* (2010), Zimmer and Fabre (2011), and Taddei and Gasser (2012)]. In this organism, chromosome organization is highly stereotypical. The 16 centromeres localize around the spindle pole body (SPB, the equivalent of the animal cell centrosome), whereas the 32 telomeres cluster in three to eight different foci at the nuclear periphery. Chromosome arms thus extend away from the SPB toward the nuclear periphery where telomeres are anchored, and their specific distribution is linked to their length. Finally, the nucleolus is positioned on the opposite side of the SPB, and is organized around 100–200 repeats of ribosomal DNA (rDNA) located in chromosome XII. Certain aspects of nuclear organization can have an impact on gene expression in budding yeast. On one hand, artificial tethering of reporter genes to subtelomeric regions and to the nuclear periphery can lead to their repression (Gottschling *et al.* 1990; Andrulis *et al.* 1998; Pryde and Louis 1999; Taddei *et al.* 2009). Moreover, perinuclear tethering of the *CLN2* cyclin gene in daughter cells mediates its repression during the G1 phase (Kumar *et al.* 2018). The association of silent information regulator (SIR) factors with telomeres also contributes to perinuclear repression (Taddei *et al.* 2009). Accordingly, genes within 20 kb of telomeres are poorly expressed, and this depends at least partially on SIR proteins and telomere anchoring to the nuclear periphery (Wyrick *et al.* 1999; Taddei *et al.* 2009). On the other hand, some inducible genes translocate from the nuclear interior to the periphery upon activation, where they interact with nuclear pore complexes (Casolari *et al.* 2004, 2005; Schmid *et al.* 2006; Taddei *et al.* 2006; Akhtar and Gasser 2007), and artificial targeting of genes to nuclear pores can also lead to their transcriptional activation (Brickner and Walter 2004; Menon *et al.* 2005; Taddei *et al.* 2006). Thus, the yeast nuclear periphery appears to harbor transcriptionally repressing and activating domains. How the three-dimensional (3D) organization of the yeast genome shapes global transcription levels remains largely unexplored.

To study the effect of nuclear organization on transcription in budding yeast, we took advantage of previously described strains bearing fusion chromosomes (FCs) (Neurohr *et al.* 2011; Titos *et al.* 2014). These cells have a grossly altered nuclear organization in interphase that is not associated with dramatic genome-wide changes in transcription, consistent with previous observations in yeast cells with extensively fused chromosomes (Luo *et al.* 2018; Shao *et al.* 2018). However, we find that displacement of FC genes away from the nuclear periphery does lead to mild, but consistent and reproducible, changes in expression across a large number of genes; on average a 10% shift away from the nuclear periphery leads to a 10% increase in expression. These effects are associated with both deletion of telomeric sequences and with displacement away from the nuclear periphery. These results suggest that radial chromosome-level spatial organization plays a limited, but significant, role in transcriptional regulation in budding yeast.

## Materials and Methods

### Polymer modeling

Each yeast chromosome of wild-type and FC strains was modeled using a bead-and-spring polymer model previously used and validated for modeling chromatin fibers (Rosa and Everaers 2008). This model consists of three different energy contributions, each describing a general physical property of the chain:

1. Excluded volume (purely repulsive Lennard-Jones potential). Each particle occupies a spherical volume of diameter equal to 30 nm and cannot overlap with any other particle in the system. Considering the typical compaction ratio of the chromatin fiber in yeast (Bystricky *et al.* 2004, 2005), each particle contains ∼3.2 kb of DNA.
2. Chain connectivity (finite extensible nonlinear elastic potential). Consecutive particles on the chain are connected with elastic potential, which allows a maximum bond extension of 45 nm. The simultaneous action of the excluded volume and the chain connectivity prevents chain crossing.
3. Bending rigidity (Kratky–Porod potential). The bending properties of an ensemble of polymer chains are usually described in terms of the *persistence length*, which is the length scale where the chain changes its behavior from rigid to flexible. According to the bending properties experimentally measured for the yeast chromatin fiber (Cui and Bustamante 2000; Bystricky *et al.* 2004; Langowski 2006), the persistence length of each model chain was set to 61.7 nm for internal regions of the chromosomes and to 195.0 nm for the terminal ones. The regions of the chains corresponding to the telomeres (the 20 kb at the chromosomes ends), in fact, are more compact and rigid (Dekker 2008).

**Figure 1:**
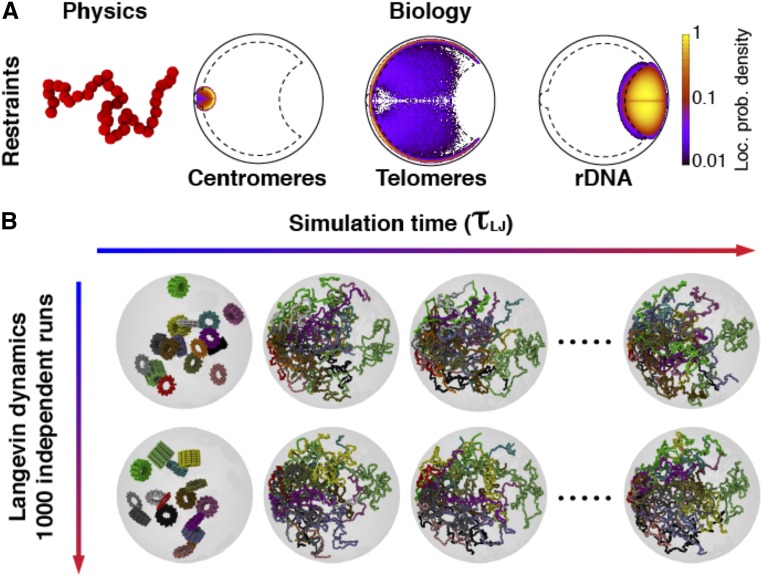
Computational modeling of the haploid budding yeast nucleus in interphase. (A) The 16 chromosomes were modeled as bead-and-spring chains with 30-nm beads each comprising 3.2 kb of DNA. The chains were confined into the nucleus (1-μm radius sphere) and beads corresponding to centromeres were constrained in a sphere of radius 150 nm attached to the nuclear envelope to mimic the attachment to SPB-mediated by microtubules. The rDNA was restrained in a region occupying 10% of the nuclear volume at the opposite side of the nucleus with respect to the SPB. The telomeres were attracted to the nuclear envelope to have higher propensity to occupy the nuclear periphery, which is defined as the spherical shell, that is the closest to the nuclear envelope and occupies one-third of the total volume of the nucleus (*Materials and Methods*). (B) The chromosomal polymer models, representing the genome-wide chromosome arrangement, were initialized as cylindrical solenoids of radius 150 nm. The solenoid chromosome states serve the sole purpose of obtaining an initial chain conformation that is compact, yet not entangled, without making any claim to reproducing any specific quantitative features of mitotic yeast chromosomes. Next, the restraints on centromeres, rDNA, and telomeric particles were satisfied using a short preliminary run of Langevin dynamics, spanning 60 τ_LJ_. Finally, the system was relaxed with a 30,000 τ_LJ_ run of Langevin dynamics, in which all the spatial restraints are in place. This run is used to obtain 10 steady-state conformations per trajectory (one every 3000 τ_LJ_). Each strain was modeled in 1000 independent replicates to obtain 10,000 genome-wide conformations per strain (*Materials and Methods*). rDNA, ribosomal DNA; SPB, spindle pole body.

Since the modeling aims to describe the chromosomal configuration of haploid strains, the total number of beads in the system is 4062, resulting from the presence of one copy of each yeast chromosome (Supplemental Material, Tables S5–S6). Each chromosome is initially folded in a solenoidal arrangement, where a rosette pattern is repeatedly stacked to yield an overall linear, rod-like conformation, see Figure 1 (Rosa and Everaers 2008; Di Stefano *et al.* 2013, 2016).

The chromosome chains are consecutively placed inside a sphere of radius 1.0 centered in the origin (0,0,0). This sphere, describing the typical shape of the yeast nucleus in G1, according to imaging data, interacts with the chromosome particles as a rigid wall. To obtain the initial chromosome nuclear locations, the positions of the chromosome centers are picked in a random, uniform way inside the nucleus, and the orientation of the rod axis is chosen randomly. The iterative placement proceeds from the longest to the shortest chromosome in a way that the newly added chromosomes must not clash with previously placed ones. In case of a clash, the placement attempt is repeated. Next, the following biological restraints (i–iii) are satisfied using a short preliminary run of Langevin dynamics, spanning 60 τ_LJ_, where τ_LJ_ is the Lennard-Jones time and is used as the time unit in Large-scale Atomic/Molecular Massively Parallel Simulator (LAMMPS):

1. To simulate the tethering of the centromeres to the SPB, the motion of the centromere particles was restrained into a spherical compartment of radius R_SPB_ = 150 nm centered in c_SPB_ = (−850,0.0,0.0).
2. rDNA particles were restrained to a region occupying 10% of the total nuclear volume and located at the opposite side of the SPB, to simulate the nucleolus. Nucleolar volume was derived from experimental measurements. This region was defined by the intersection of the nuclear sphere with a sphere of radius R_NUCL_ = 640.92 nm whose center is located at c_NUCL_ = (1000,0.0,0.0). Conversely, the other no-rDNA particles of the chromosome models were restrained to stay out of the same nucleolar region.
3. Finally, to represent the tendency of the telomeres to stay anchored to the nuclear envelope (NE), the periphery of the sphere (a shell within R_PER_ = 126 nm from the NE, which accounts for one-third of the nuclear volume) was set to be attractive for the terminal particles of the chromosome chains. This effect, unexplored so far, was accomplished using a Lennard-Jones attraction (Jones 1924). It is important to note that telomeres were not strictly confined at the nuclear periphery of our models, but they were only favored to be close to the NE using a short-range interaction, which could be overcome by forces acting in the telomeres of chromosomes. Indeed, the first and last beads of each chromosome (telomeres) were not always peripheral in our simulations. If the nucleus was divided into three concentric spherical shells of the same volume, telomeres occupied the medium or central parts of the nucleus in ∼40% of the models, as shown in Figure S7.

The restraints listed above were imposed, applying on each of the involved particles a force F, only when the particle did not satisfy the confinement conditions, using the option indent of the software LAMMPS (Plimpton 1995):

$$F\left(r\right)\mathrm{=-10}{\left(\mathrm{r-R}\right)}^{2},$$

where r is the distance from the particle to the center of the sphere and R is the radius of the sphere.

In the FC strains, the chromosomes involved in the fusion were attached to each other using additional connectivity bonds (chain connectivity in point 2 above) between the telomeres involved in the fusion process. These telomeres, which were attracted to the periphery in the wild-type strain models, behaved as internal chromosomal sequences in the FC strains and lost the telomeric attraction to the NE.

Finally, the system was relaxed using a run of Langevin dynamics of 30,000 τ_LJ_, and one conformation every 3000 τ_LJ_ (10 models per trajectory) was retained for analysis. Replicating the complete simulation 1000 times generated 10,000 genome-wide conformations per strain.

### Analysis of the genome-wide models and calculation of changes in % peripheral

Various measures were performed to characterize the generated structural models:

1. Building on the representations in Tjong *et al.* (2012), two-dimensional (2D) localization probability density plots of chromosomes were generated. For each chromosome, the Cartesian coordinates (*x*, *y*, *z*) of the particles were collected and then projected into a 2D reference frame made of an axial coordinate (along the SPB-to-nucleolus direction of the model nucleus that is *x*-axis in this work) and a radial one: (a, ρ)=(x, $\sqrt{y^{2}+z^{2}}$). In the 2D (a, ρ) plan, the points are represented in a grid to produce the final heatmap. The grid size was 2 × 2 µm and the cell dimension was 10 nm. Once a point (a_c_, ρ_c_) is mapped onto the grid, since the particle is larger than the pixel of the grid, a Gaussian blur (σ=30nm) is applied centered at the corresponding pixel. The values of the heatmap are finally normalized from 0 to 1 (Figure 1A and Figure 4).
2. To characterize the nuclear positioning of each locus, the volume of the model nucleus was divided into three concentric shells, each spanning one-third of the total nuclear volume. In each simulation, all chromosome particles (3.2-kb loci) were next categorized as central, middle, or peripheral depending on which of the three shells they occupied. This measure was used to generate the plots of the predicted percentage in periphery per particle and the percentage of shell occupation per terminal (telomeres) particle (Figure S9). The latter quantities were averaged over the ensemble of 10,000 model conformations.
3. By mapping the annotated genes on the 3D models, the predicted percentage in the periphery for each gene was computed as the average of the constitutive particles. Subtracting the percentage in periphery computed in the wild-type to the value in the FC strain and taking the absolute value, the decrease in percentage in the periphery was then calculated (Figure 7).
4. The “displacement from NE” and the “difference in distance to the NE” the for 10-kb regions of the models were computed as follows. First, the distance between each particle and the NE was computed for each strain. Next, each chromosome was partitioned in groups of three consecutive particles (which correspond to about 10 kb) and the distance of each 10-kb locus from the NE was computed as the average distance of the particles within the locus. The displacement and the difference in the distance were then computed comparing the FC strains to the wild-type one (Figure 5).
5. The genomic locations of the *LYS4* and *TRP4* genes were mapped on the models, and the distances from the NE (or from SPB or between the two genes) were computed as the average of the corresponding particle-based distances (Figure 6).
6. The contact maps were computed for the wild-type strain using a distance cutoff between particles of 120 nm and binning at 32 kb (corresponding to 10 model particles) of resolution. The 3C (Chromosome Conformation Capture) interaction maps (used for model validation, not as input for modeling) were obtained by downloading the data sets from Duan *et al.* (2010) obtained using the *Hin*dIII restriction enzyme and the raw reads from Gene Expression Omnibus (GEO) accession number SRR5077790 from Lazar-Stefanita *et al.* (2017). The latter was next analyzed using the TADbit (Serra *et al.* 2017) pipeline to obtain the raw interaction maps and the OneD procedure (Vidal *et al.* 2018) to normalize it (Figure S1).
7. The median telomere–telomere (terminal particle of the chromosome model) distance was computed for each of the 60 telomere pairs considered in Therizols *et al.* (2010) (Figure S2) and correlated with the experimental measures performed therein.
8. The displacement of model particles from the SPB in Figure S4 was computed as follows: (i) for each strain and all model conformations, the distances between each particle individually and the SPB were computed; (ii) the average particle–SPB distances were computed for each particle in each strain; (iii) for each FC strain, the difference in the (average) distance to the SPB with respect to the wild type were computed for each particle; and (iv) the difference was computed such that positive values indicated a (typical) displacement away from the SPB in the FC strains and negative values indicated displacement toward the SPB in FC strains.

### Previously published modeling approaches

The *S. cerevisiae* genome has been previously modeled using two main restraint-based approaches. First, 3C data sets have been used as input restraints to reconstruct the 3D conformation of the yeast genome (Duan *et al.* 2010; Lesne *et al.* 2014; Lazar-Stefanita *et al.* 2017). Second, and in a similar approach to that used in our work, models were built using genome tethering to nuclear elements as restraints (Tjong *et al.* 2012; Wong *et al.* 2012). The differences between our approach and these previously published studies are minimal. For example, in this work, the genome was represented as a series of spherical beads compared to cylinders previously used by Wong *et al.* (2012). Moreover, the initial conditions of the simulation, the confinement of the genome, and the minimization protocols were different in our work compared to those used by Tjong *et al.* (2012). However, these differences are likely to minimally change the final conclusions of our modeling approach compared to those previously published.

### Strains, cell growth, and microscopy

*S. cerevisiae* strains are derivatives of S288c. TetO/LacO cells and chromosome fusions were previously described. Briefly, FC chromosomes were obtained by successive rounds of homologous recombination between subtelomeric regions of two chromosomes, by transformation of haploid yeast cells with a PCR-generated DNA fragment containing a resistance cassette flanked by sequences homologous to the subtelomeric regions of two different chromosomes. Formation of dicentric chromosomes was avoided through activation of a *GAL1,10* promoter inserted next to centromere 4 and selection of FC recombinants in galactose. When fusing three or four chromosomes, one of the centromeres was deleted and fusion with another chromosome was repeated. To allow the reuse of selection markers, the *URA3* cassette was deleted by homologous recombination and ura- recombinants were selected on 5-FOA. Finally, the conditional *CEN4* locus was deleted or replaced with a wild-type copy to ensure robust growth in glucose. Strains were confirmed by PCR, and by the segregation timing of *TRP1* and *LYS4* loci by time-lapse imaging, as previously described in detail (Neurohr *et al.* 2011; Titos *et al.* 2014). Live-cell microscopy was carried out on a confocal spinning disk (Nikon, Garden City, NY) equipped with an HCX plan APO 100X objective and a Photometrics Prime 95B camera. Eleven 0.2-μm thick z-sections were collected. Distances were measured between local maxima (*i.e.*, the brightest pixels of fluorescent spots or the center of the nuclear rim) on single planes using ImageJ (http://rsb.info.nih.gov/ij/), although for clarity, figures are represented as 2D maximum projections of whole-cell Z-stacks. Graphs and statistical analysis (Student’s *t*-test allowing for unequal variance) were performed with R and Excel (Microsoft).

### Immunofluorescence and FISH

To make FISH probes, a 6-kb PCR fragment in the *TEL4R* region was amplified from genomic DNA with primers: 5′-ATCTTTCCTTACACATAAACTGTCAAAGGAAGTAACCAGG-3′ and 5′-GTAACATACAAACTCAACGCCTACTAAGATTAATACATCA-3′, and labeled with Alexa Fluor 488 by nick translation using the FISH Tag-DNA Multicolor Kit (Invitrogen, Carlsbad, CA). FISH-immunofluorescence was performed essentially as described (Gotta *et al.* 1999), with minor modifications. First, 1–2^9^ cells of exponential cultures (OD_600_ = 0.5–1) were collected, resuspended in 500 μl of 0.1 M EDTA/KOH pH 8.0 and 10 mM DTT, and incubated for 10 min at 30°. Cells were collected and resuspended in 0.1 M KPi (pH = 6.4)/1.2 M sorbitol and digested with 0.4 mg/ml Zymolyase 100T (Seikagatu) for 5–15 min at 30° in 0.1 M KPi (pH = 6.4)/1.2 M sorbitol. This treatment allowed the cells not to be completely converted into spheroplasts, but instead partially retain their cell walls, to help stabilize their 3D structure. Partially spheroplasted cells were fixed for 20 min with 3.7% paraformaldehyde in YPD/1.2 M sorbitol at room temperature. Cells were recovered by centrifugation (1000 × *g* for 5 min), washed three times in YPD/1.2 M sorbitol, resuspended in 0.1 M KPi (pH = 6.4)/1.2 M sorbitol, and spotted on Teflon slides; after being left to air-dry for 5 min, they were immersed in cold methanol for 6 min and in cold acetone for 30 sec. Slides were then rinsed in PBS containing 0.1% Triton X-100 (PBS-T) and 1% BSA, and incubated for 30 min at room temperature. Spots of the slide were dried and incubated overnight at 4° (or for 1 hr at 37°) with anti-Nuclear Pore Complex antibody (Mab414, ab24609, Abcam), diluted 1:2 in PBS-T 1% BSA. Slides were then washed in PBS-T and incubated with anti-mouse Alexa 647 (A-21236, Life Technologies) diluted 1:200 in PBS-T and 1% BSA at 37° for 1 hr. Next, slides were fixed again in PBS containing 3.7% paraformaldehyde for 20 min and incubated overnight in 4× SSC, 0.1% Tween 20, and 20 μg/ml of RNase A at room temperature. Slides were then washed in water, sequentially immersed for 1 min in 70, 80, 90, and 100% ethanol at −20°, and air-dried. Slides were then denatured at 72° with 70% formamide and 2× SSC, immersed for 1 min sequentially in 70, 80, 90, and 100% ethanol at −20°, and air-dried. The hybridization solution (50% formamide, 10% dextran sulfate, 2× SSC, 0.05 mg/ml labeled probe, and 0.2 mg/ml single-stranded salmon sperm DNA) was then applied and slides were incubated at 10 min at 72°. Slides were incubated for 48 hr at 37° to allow probe hybridization, and washed twice for 10 min each at 42° in 0.05× SSC and twice in buffer (0.15 M NaHCO_3_ and 0.1% Tween 20, pH 7.5) with 0.05% BSA for 30 min. After three washes in BT buffer, 2 μl of DAPI (Roche Diagnostics) 2.5 mg/ml were added and incubated for 1 min. Slides were washed twice with 0.05× SSC and mounted in 1× PBS, 50% glycerol, and 24 μg/ml 1,4diazabicyclo-2,2,2,octane, pH 7.5.

### RNA sequencing

Cells were harvested by centrifugation and RNA was extracted from fresh pellets using the RiboPure Yeast Kit (Ambion). RNA concentrations were determined using a NanoDrop 1000 (Thermo Scientific), while quality and integrity were checked using a Bioanalyzer 2100 (Agilent Technologies). RNA sequencing (RNA-seq) was performed on a HiSeq2000 (Illumina). Paired-end reads of 50 bp were aligned to the reference *S. cerevisiae* genome (R64-1-1) using kallisto quant -i orf_coding_all.idx -o output -b 100 read1_file.fastq.gz read2_file.fastq.gz.

To obtain a robust and accurate wild-type expression level for each gene, we averaged across strains. For each strain in which the gene was predicted to increase or decrease time spent in the nuclear periphery by < 1%, we took the median expression value across all strains (four independent RNA-seq replicate experiments per strain). Fold-change in expression was calculated as the log2 ratio of expression in the FC strain divided by expression in this median expression value. Similar results were obtained if expression for the wild-type control strain was used, but as many of the genes were expressed at very low levels, and hence represented by very few reads, averaging across strains was more robust to random counting noise.

### Data availability

Yeast strains are available upon request. Data and codes are available at https://github.com/Lcarey/DiGiovanni_DiStefano_FC. RNA-seq raw data are available at https://www.ncbi.nlm.nih.gov/geo/query/acc.cgi?acc=GSE108261 with GEO accession Nr GSE108261. Supplemental material available at figshare: https://doi.org/10.25386/genetics.11516508.

## Results

### A computational model to study the impact of yeast nuclear organization in gene expression

To study how the 3D organization of the genome affects gene expression, we first sought to establish how gene position correlates with transcription levels in wild-type budding yeast cells. To estimate gene position, we built computational models of chromosomes in the interphase G1 nucleus, a strategy that has proven useful in recapitulating chromosome-level nuclear organization in budding yeast (Tjong *et al.* 2012; Wong *et al.* 2012; Dultz *et al.* 2016). We modeled chromosomes as bead-and-spring chains, an approach previously validated for modeling the general physical properties of chromatin fibers (Rosa and Everaers 2008; Di Stefano *et al.* 2013). Details of the polymer modeling process are found in the *Materials and Methods* and summarized in Figure 1A. Briefly, chromosomes were confined inside a sphere of 2 µm diameter corresponding to the interphase nuclear size. Centromeres were confined to a spherical region of radius 150 nm at one pole of the nuclear sphere to account for the tethering of centromeres to the SPB by microtubules (O’Toole *et al.* 1999). The dynamic association of telomeres with the NE was modeled with the periphery of the sphere attracting the terminal beads of chromosome chains. Finally, to reproduce the confinement of the rDNA in the nucleolus, the particles corresponding to rDNA were restrained to a region located at the opposite side of the SPB. An ensemble of chromosomal polymer models was generated using Brownian motion dynamics. A total of 10,000 model conformations satisfying all the imposed restraints were then selected, and analyzed for the likelihood of particular loci and chromosomes being positioned in specific regions of the cell nucleus (Figure 1B).

As an orthogonal validation of our model, we compared the probability of contact among all chromosomal particles in the wild-type models with the experimentally measured intra- and interchromosomal contact frequencies observed by a 3C-derived technique (Duan *et al.* 2010; Lazar-Stefanita *et al.* 2017). Specifically, we compared the internal correlations between models’ and experimental contact matrices (Imakaev *et al*. 2012) and the correlations between matrix elements grouped by genomic distance (Figure S1B and C) and found in both cases significant similarities between models and experiments. In addition, we compared the predicted median telomere–telomere distances from our models with analogous experimental data obtained using imaging (Therizols *et al.* 2010). In both comparisons, we found that our models, based on the physical properties of chromatin and minimal biological restraints, accurately described wild-type yeast nuclear organization (Figures S1–S2). This confirms the validity of polymer-based modeling to reproduce nuclear organization features (Tjong *et al.* 2012; Wong *et al.* 2012).

**Figure 2:**
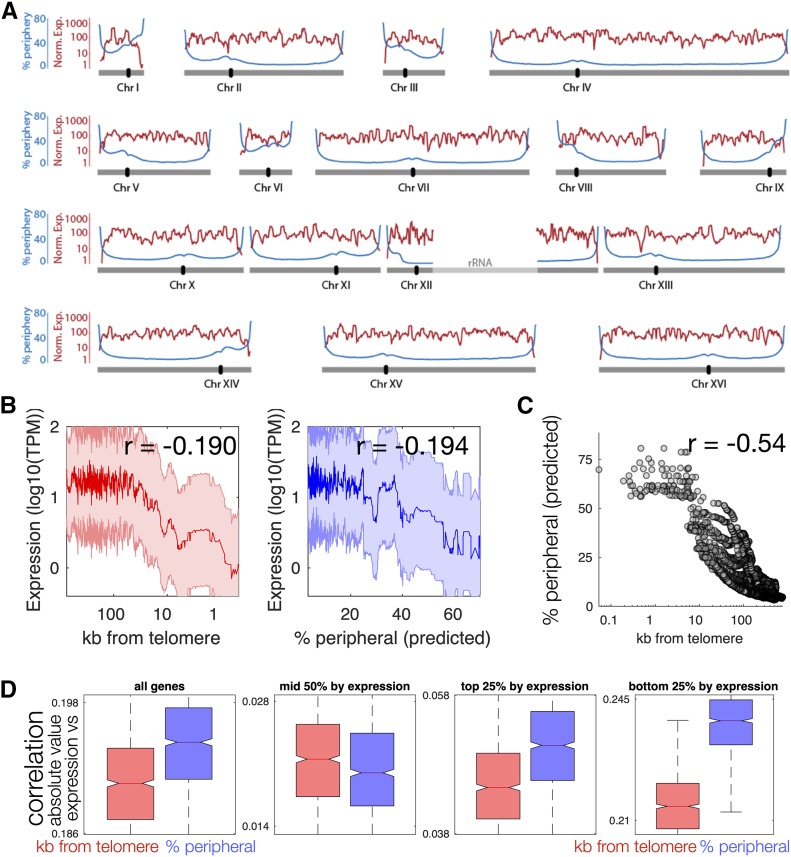
Localization in the nuclear periphery is associated with lower expression. (A) mRNA expression (red) and predicted time spent in the nuclear periphery (blue) are shown for each chromatin bead along each of the 16 yeast Chrs. (B) Median expression level for genes binned by distance to the telomere (red) or by predicted % peripheral (blue). Correlation values are for Pearson correlation on unbinned data. (C) Predicted % peripheral is not perfectly correlated with distance from the telomere. (D) The *y*-axis values are absolute values of the correlation of either kb from the telomere (red) or % peripheral (blue), with gene expression. The two distributions (red and blue) are generated by randomly sampling the data 1000 times. Across all but one grouping, gene expression is more strongly correlated with predicted % peripheral (blue) than with log(distance to the telomere) (red). The difference is larger with linear distance to the telomere (not shown). Boxplots show median correlation across 1000 random samplings of 90% of genes. All distributions are significantly different from each other due to the large number of computational samplings. The statistical differences and effect size are largest in genes in the bottom 25% of expression. Chr, chromosome; mRNA, messenger RNA; TPM, transcripts per million.

To determine if our computational models were consistent with the experimentally measured low gene expression at the nuclear periphery, the predicted gene position relative to the nuclear periphery was correlated with genome-wide messenger RNA (mRNA) levels obtained by RNA-seq. Genomic regions within 30 kb of the ends of wild-type chromosomes were poorly expressed, consistent with previous reports (Wyrick *et al.* 1999) (Figure 2, A and B). Importantly, lower expression was also correlated with gene peripheral localization, as predicted by polymer modeling (Figure 2B). Because most subtelomeric sequences are also restricted to the perinuclear region, the above analysis confounds the contributions of sequence proximity to chromosome ends [one-dimensional (1D) effect] and proximity to the nuclear periphery (3D effect) to steady-state mRNA levels. However, we found that, while distance to the telomere and predicted location in the nuclear periphery were correlated, they were imperfectly so (Figure 2C). Especially for genes with low expression, the fraction of modeled nuclei in which a gene was predicted to be at the nuclear periphery was more highly correlated with expression than distance to the telomere in both linear (correlation = −0.093) and log space (Figure 2D and Figure S3**)**. Furthermore, in a linear model that predicts expression from both of the two variables, % peripheral is a slightly more important feature (Table S1). These data open the possibility that localization to the periphery, and not only distance from the telomere, is partially responsible for low expression.

### Computational modeling and cell imaging validate nuclear reorganization after chromosomal rearrangements

**Figure 3:**
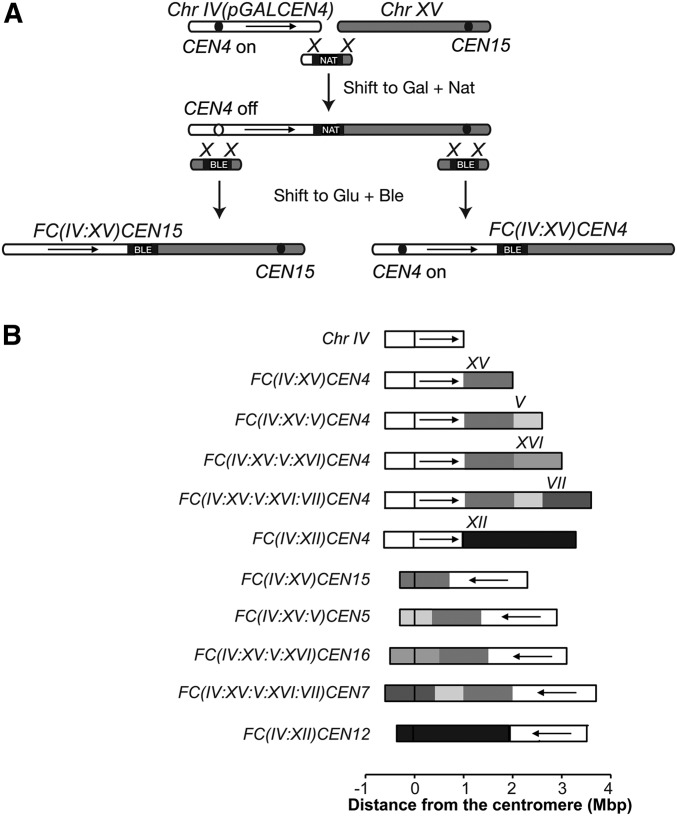
Generation of FC strains. (A) The generation of FCs [originally described in Neurohr *et al.* (2011) and Titos *et al.* (2014)] starts with the integration of *pGAL1* sequence upstream of the centromere to be inactivated. Next, the Chrs are fused by homologous recombination between a bridging PCR fragment and the telomeres of the Chrs. Finally, the deletion of one of two centromeres and the excision of the *pGAL1* sequence, as appropriate, generates the *FC* strain. Black circle is the centromere, black rectangle is the selection marker. (B) Schemes of all the *FC* strains used in this work. Chr IV is shown for comparison. Arrows indicate the relative orientation of this Chr in the fusions. Ble, bleomycin resistance cassette; Chr, chromosome; FC, fused chromosome; Gal, galactose; Glu, glucose; Nat, nourseothricin resistance cassette.

To experimentally determine if spatial organization affects expression, we next examined how large-scale chromosome rearrangements affect nuclear reorganization. In previously described FC strains, up to three “donor” chromosomes were sequentially fused to the end of a “recipient” chromosome (Neurohr *et al.* 2011; Titos *et al.* 2014). Centromeres were simultaneously removed from donor chromosomes to avoid formation of toxic dicentrics; telomere elements at the site of the fusion were also removed. Thus, like normal chromosomes, FCs contain two telomeres and one centromere (Figure 3, A and B). These chromosome fusions only minimally changed the genomic content relative to wild-type strains, since only 5 to 26 subtelomeric ORFs are lost during the fusion procedure (Table S2). However, we hypothesized that FC strains would display dramatically altered interphase chromosome organization. Indeed, this is dependent on chromosome number and length, centromere attachment to SPBs, and telomere anchoring to the NE, all of which are altered in FC strains. Importantly, chromosome fusions led to a maximal reduction in chromosome and centrosome number from 16 to 13, reduction of telomere number from 32 to 26, and lengthening of the longest chromosome arm (excluding chromosome XII, containing the variable rDNA array) from 1 to almost 4 Mb (Figure 3B).

We then applied the principles used in modeling wild-type nuclei to determine nuclear organization in the 10 different FC strains (Figure 3B). FCs used in this study are named using the following convention: FC is followed by the chromosomes that comprise the fusion indicated in brackets, followed by the centromere of the recipient chromosome. Thus, strain *FC(IV:XV:V)CEN4* bears an FC in which chromosome IV is the recipient, and chromosomes XV and V are the donors.

**Figure 4:**
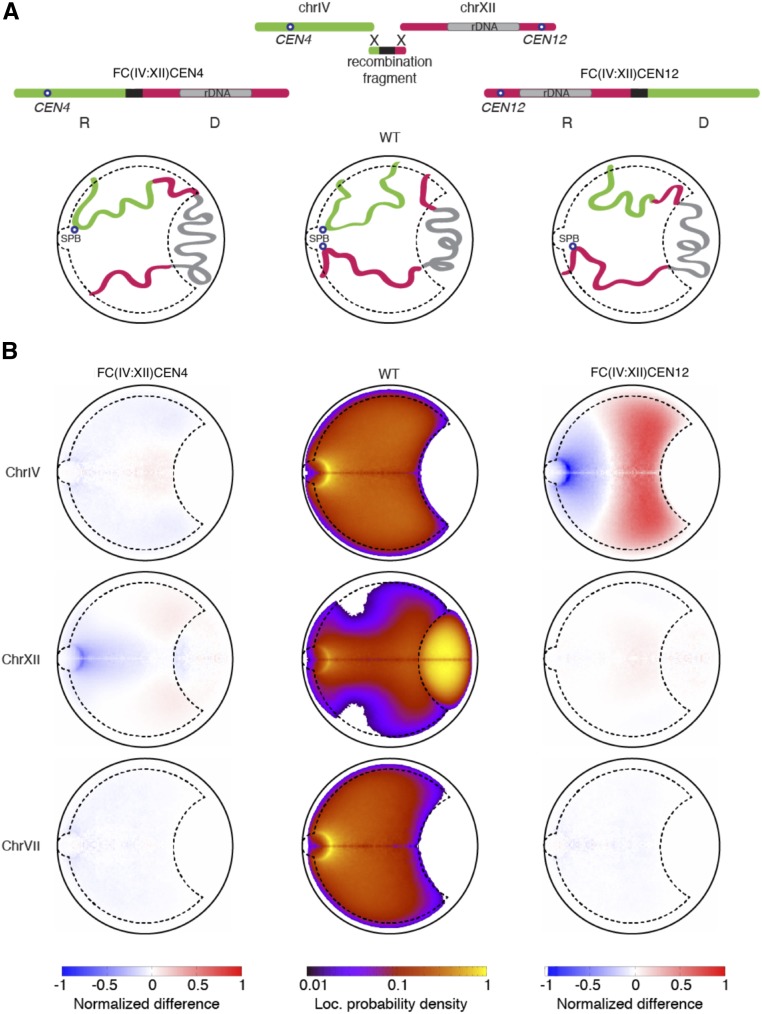
The donor Chrs are predicted to be strongly displaced in the nucleus. (A) Cartoon representations of WT, *FC(IV:XII)CEN4*, and *FC(IV:XII)CEN12* strains. “Donor” and “recipient” Chrs are labeled “D” and “R,” respectively. (B) Predicted Chr loc. probability densities for Chrs IV, XII, and VII in the wild-type strain (central column), and the *FC* strains *FC(IV:XII)CEN4* (left column) and *FC(IV:XII)CEN12* (right column), shown normalized by the WT strain. The heatmaps show large differences in the positioning of the recipient and donor Chrs, and almost no difference in the nuclear organization of the largest nonfused one, Chr VII. Chr, chromosome; FC, fused chromosome; loc., location; rDNA, ribosomal RNA; WT, wild-type.

The model predicts two major changes in the FC strains. First, large (> 300 nm) displacements of donor chromosomes away from the SPB and slight (10–20 nm) displacement of recipient chromosomes toward the SPB (Figure 4 for IV:XII fusions, and Figure S4 for all FCs). Both of these displacements can be interpreted as a consequence of the deletion of centromeres in donor chromosomes. Indeed, centromere deletion removes the anchoring of donor chromosomes to the SPB, while also reducing chromosome density close to the SPB. Thus, abnormally large FCs will tend to occupy the space far from the SPB, whereas remaining centromeres will be allowed to occupy positions closer to the SPB. The combined action of these phenomena induces an effective pressure on the recipient chromosomes, which are pushed closer to the SPB compared to in the wild-type scenario (Figure S4).

**Figure 5:**
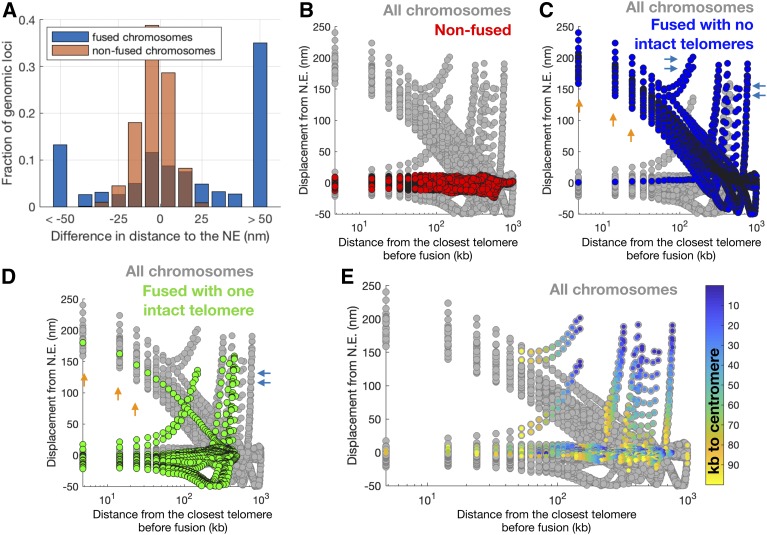
Loci predicted to be displaced away from the nuclear periphery are near centromeres and telomeres of fused chromosomes. (A) The predicted displacement with respect to the N.E. for loci of 10 kb in fused (blue) and nonfused (orange) chromosomes. Brown is the superposition of blue and orange. Only loci on fused chromosomes are displaced from the nuclear periphery. (B–E) The predicted displacement from the N.E. (*y*-axis) as a function of the distance from the telomere before chromosome fusion (*x*-axis). Each circle represents a 10-kb locus in the model. Models of all FC strains are included. In each panel, circles corresponding to a subset of chromosomes are colored, whereas the rest are gray. In (B–D), chromosomes are color-coded as “nonfused” (red), “fused with no intact telomeres” (blue), and “fused with one intact telomere” (green). For example, chromosome XV is fused with no intact telomeres in FC(IV:XV:V) strains, and fused with one intact telomere in FC(IV:XV) strains. (B) Loci in nonfused chromosomes are not displaced relatively to the N.E. (all values are near zero). (C) Loci in fused chromosomes that have both telomeres engaged in fusion events show two types of displacement. Most loci that are displaced away from the periphery are subtelomeric (orange arrows), indicating that subtelomeres that participate in fusions lose attachment to the nuclear periphery. However, some nonsubtelomeric loci are also displaced (blue arrows). (D) Loci in fused chromosomes that have only one telomere engaged in fusion events also show two types of displacement. Some subtelomeric loci are displaced from the periphery (presumably those that participate in a fusion event; orange arrows). Some nonsubtelomeric loci are also displaced (blue arrows). (E) Loci are colored not according to their location in fused or nonfused chromosomes, but according to their distance to a centromere before fusion. This shows that all noncentromeric loci that are displaced away from the periphery in (B–D) were pericentromeric before the fusion occurred. N.E., nuclear envelope.

Second, the model predicts displacement of loci in the fused chromosomes away from the nuclear periphery, as shown in Figure 5. To quantify this prediction, we computed the distance from the nuclear periphery of all 10-kb loci from the surface of the nuclear sphere for all chromosomes in all strains relative to wild-type. The model predicts that only loci on fused chromosomes are displaced away from the nuclear periphery, while the relative location of loci in nonfused chromosomes never varies by >50 nm (Figure 5A). Loci with the largest predicted displacement were located near telomeres (Figure 5, B–D) or centromeres (Figure 5E) before the fusion event. These displacements can be interpreted as a result of the deletion of centromeres and telomeres in fused chromosomes, as these elements provide anchoring to the SPB and the NE, respectively.

**Figure 6:**
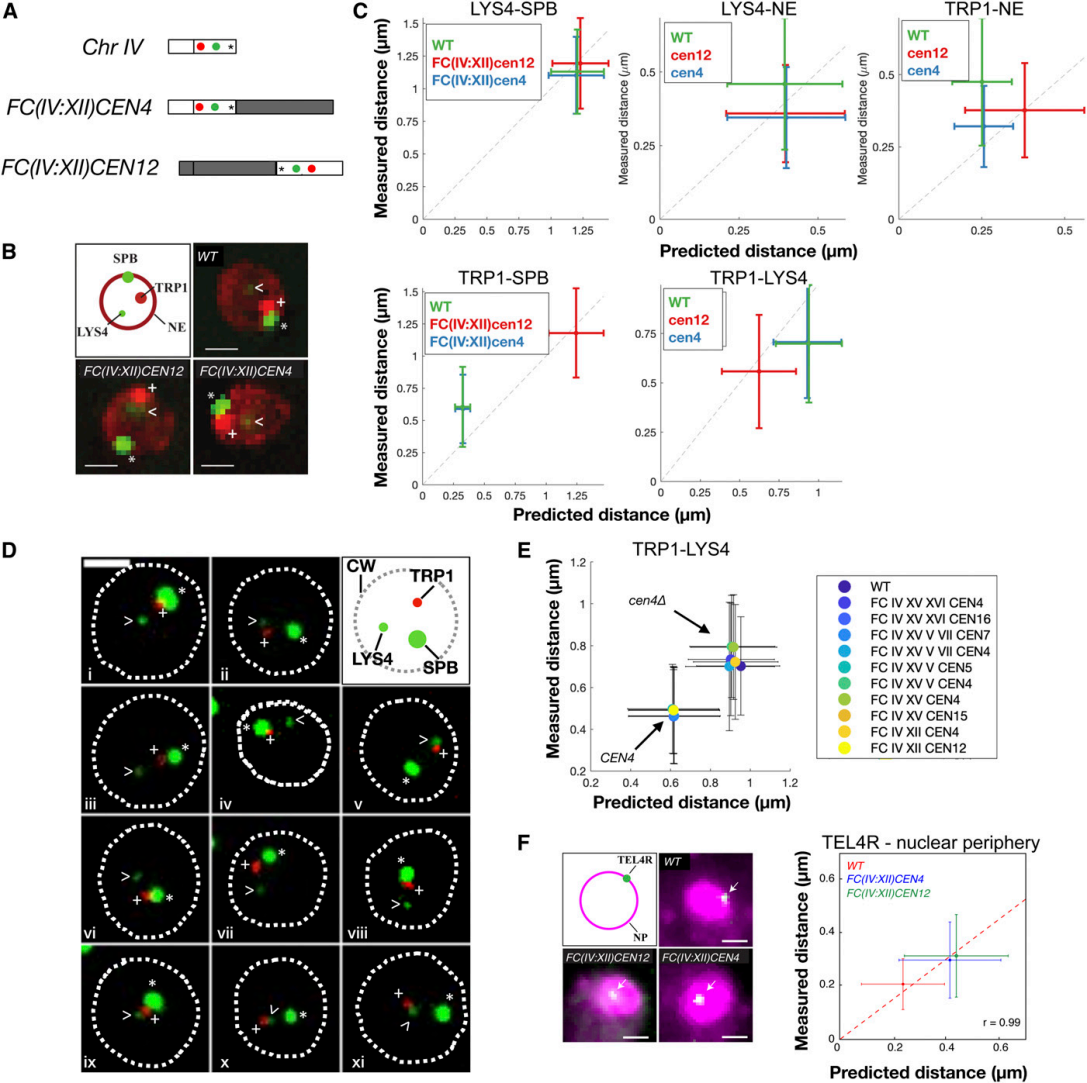
Validation of polymer models by live- and fixed-cell microscopy. (A) Position of *TRP1* (red), *LYS4* (green), and *TEL4R* (asterisk) on Chr IV and its indicated FC derivatives. (B) Live-cell microscopy of G1 cells of the indicated strains showing the localization of *TRP1* (red dot, marked with +), *LYS4* (faint green dot, arrowhead), the SPB (bright green dot, marked with an asterisk), and the nuclear periphery labeled with Nup49-mCherry (red). (C) Correlation of measured and predicted distances between the indicated nuclear loci, the SPB, and the nuclear periphery in the indicated strains. Graphs show the means and SDs for WT (151 cells), *FC(IV:XII)CEN4* (152 cells), and *FC(IV:XII)CEN12* (153 cells), and 10,000 independent simulations. (D) Live-cell microscopy of G1 cells of the indicated strains showing the localization of *TRP1*, *LYS4* and the SPB marked as in (B). Note that the NE is not labeled and the dotted line indicates the CW. Strains shown are: (i) *FC(IV-XII)CEN4*, (ii) *FC(IV-XII)CEN12*, (iii) *WT*, (iv) *FC(IV-XV)CEN4*, (v) *FC(IV-XV)CEN15*, (vi) *FC(IV-XV-V)CEN4*, (vii) *FC(IV-XV-XVI)CEN4*, (viii) *FC(IV-V-VII-XV)CEN4*, (ix) *FC(IV-XV-V)CEN5*, (x) *FC(IV-XV-XVI)CEN16*, and (xi) *FC(IV-V-VII-XV)CEN7*. Bar, 2 μm. (E) Correlation of measured and predicted distances between *TRP1* and *LYS4* in the indicated strains. Graphs show the means and SDs for simulations (10,000 iterations) and experimental data (>100 cells for each strain). Distances are shorter in strains in which Chr IV acts as a donor (*cen4*∆) compared to Chrs in which it acts as a recipient of fusions (*CEN4*). (F) FISH of G1 cells of the indicated strains showing the localization of *TEL4R* (green dot, arrows) and the nuclear periphery labeled with DAPI (magenta). Graph shows the means and SDs from WT (95 cells), *FC(IV:XII)CEN4* (82 cells), and *FC(IV:XII)CEN12* (102 cells), and 10,000 independent simulations. Bar, 1 μm. Chr, chromosome; CW, cell wall; FC, fused chromosome; NE, nuclear envelope; SPB, spindle pole body; WT, wild type.

To validate predicted chromosome displacement in FC strains, we determined the distances of chromosome loci to each other, to the SPB, and to the nuclear periphery using fluorescence microscopy in wild-type and FC strains during G1. Loci in chromosome IV were visualized through Tet repressor fused to monomeric red fluorescent protein (TetR-mRFP) and Lac inhibitor fused to green fluorescent protein (LacI-GFP) reporters in cells bearing tetracycline and lactose operator arrays. These arrays were inserted, respectively, at the *TRP1* locus 10-kb away from *CEN4* in the right arm of chromosome IV and at the *LYS4* locus in the middle of the chromosome IV right arm, 470-kb away from *TRP1*. Spc42-GFP and Nup49-mCherry were used to label SPBs and the nuclear periphery, respectively. We first determined distances between these nuclear landmarks in wild-type and in the two FC strains *FC(IV:XII)CEN4* and *FC(IV:XII)CEN12* (see scheme in Figure 6A). We then compared these measured distances with model predictions. Measured and predicted distances were significantly correlated for all distances across the examined strains (Figure 6, B and C). Neither *TRP1* nor *LYS4* changed their distances from the nuclear periphery in either FC, consistent with model predictions. In contrast, the *CEN4*-associated *TRP1* locus was located in the vicinity of the SPB in wild-type and *FC(IV:XII)CEN4* nuclei, whereas the same locus was displaced away from the SPB in *FC(IV:XII)CEN12* (Figure 6C). This is consistent with the mitotic segregation timing (relative to spindle elongation) of these FCs (Neurohr *et al.* 2011; Titos *et al.* 2014) and with model predictions that donor chromosomes are displaced away from the SPB, as compared to the wild-type configuration. Furthermore, we observed that the distance between *TRP1* and *LYS4* was reduced in the *FC(IV:XII)CEN12* relative to wild-type and *FC(IV:XII)CEN4* cells, and that this was in agreement with the models (Figure 6C). Shortening of *TRP1-LYS4* distances was observed in all FC strains in which chromosome IV acted as a donor (Figure 6D), and again, this was in agreement with the models (Figure 6E). These observations suggest that displacement of a genomic region away from an active centromere and/or to a nuclear region away from the SPB leads to its increased compaction. This could be due to elimination of microtubule-dependent pulling forces on the neighboring kinetochore and/or to increased chromatin crowding in SPB-distal nuclear regions. Finally, FISH established that the *TEL4R*-proximal locus was closely associated with the nuclear periphery (labeled with DAPI) in wild-type cells, whereas the mean distance between *TEL4R* and the nuclear periphery was increased in both *FC(IV:XII)CEN4* and *FC(IV:XII)CEN12* fusions (Figure 6F). Because the *TEL4R* region is engaged in chromosome fusions in all FC strains (Figure 3B), this region is most likely displaced in these strains as well. This confirmed the model’s prediction that subtelomeric loci engaged in a chromosome fusion event are displaced away from the periphery (Figure 5, B–D). Together, these results quantitatively confirm the model predictions that chromosome fusions lead to large changes in the subnuclear organization of chromosome regions relative to each other, to the SPBs, and to the nuclear periphery.

### Chromosomal rearrangements reveal a correlation between increased expression and gene displacement from the nuclear periphery

To determine whether the genome reorganization caused by chromosome fusions led to changes in gene expression, we performed RNA-seq in the 10 FC strains (Figure 3), with four independent RNA-seq replicate experiments per strain. Consistent with all FC strains having wild-type growth rates (Neurohr *et al.* 2011; Titos *et al.* 2014), the presence of FCs did not correlate with strong changes in gene expression (Figure S5**)**. This suggests that spatial chromosome displacements (such as changes in gene location relative to the SPB and to other chromosomes) do not strongly affect gene expression.

**Figure 7:**
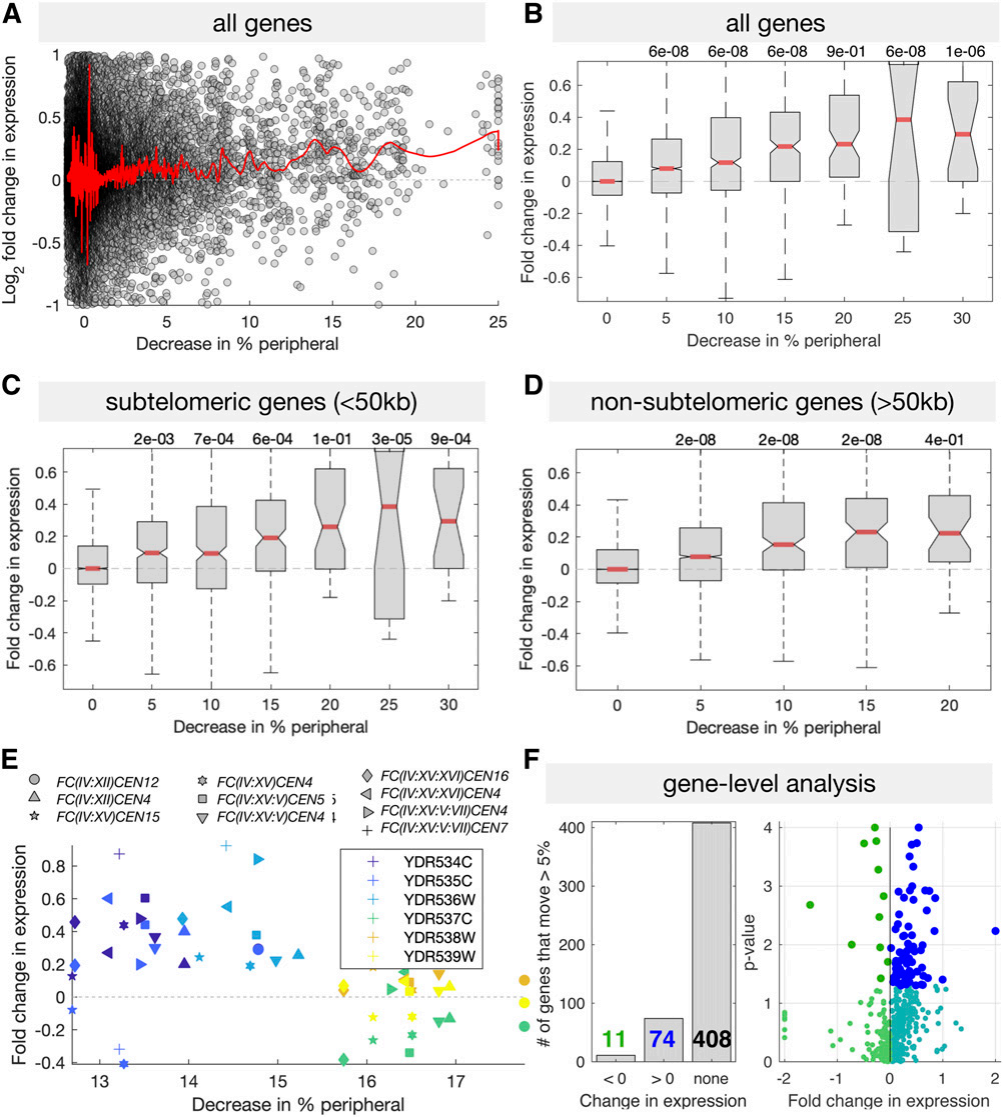
Gene displacement away from the nuclear periphery correlates with increased expression. (A) Shown for all genes and all strains are the fold changes in expression, and changes in the predicted localization to the nuclear periphery. Red line shows a LOESS fit with a window size of 100 genes (MATLAB smooth() with the “rloess” option). (B–D) The same data as in (A), with genes grouped by the predicted decrease in peripheral localization. (E) Measured expression and predicted change location for the six genes around TEL4R, which are shown to be displaced from the periphery in Figure 6F. Colors mark genes and symbols mark strains. This region is predicted to be in the periphery, has ∼15% fewer nuclei, and all genes save YDR537C increase in expression. (F) The number of genes predicted to move >5% that exhibit significant (Student’s *t*-test *P* < 0.05) changes in expression. (B–D) *P*-values are tests for difference in the mean between each group and the nondisplaced group, using Tukey’s honestly significant difference criterion to correct for multiple hypothesis testing, using anova1() and multcompare() in MATLAB. LOESS, locally estimated scatterplot smoothing.

We then asked whether mild changes in expression correlated with changes in predicted gene position relative to the nuclear periphery. Gene expression analysis was performed with four biological replicates for wild-type, and three or four biological replicates for each of 10 FC genotypes, for a total of 42 RNA-seq experiments. To calculate differential expression we compared the median expression in the four replicates of wild-type to the median expression for all replicates of each FC strain. Thus, for each gene, we obtained 10 differential expression values. As each FC strain was also modeled separately, we generated a matched set of 10 predicted changes in location. Thus, each gene in each FC strain was a point defined by its measured change in expression and its predicted change in location. To obtain a more accurate value for expression in the absence of changes in nuclear location, for each gene we used the average expression level of that gene across all strains in which the percent peripheral was not predicted to increase or decrease by > 1%. From this baseline expression value, we compared the fold change in expression for each strain with the predicted change in the frequency with which each gene was located in the nuclear periphery. Genes deleted during the fusion events were not considered. The results of this analysis show mild but statistically significant genome-wide expression changes for genes that change location relative to the nuclear periphery after chromosomal fusions (Figure 7, A and B). For example, the median gene with a predicted 25% decrease in association with the periphery due to chromosome fusion exhibits a 25% increase in expression in FC strains (Figure 7B). While the effect on expression is weak, it is consistent across changes in localization and strains, and remains if we limit our analysis to genes not involved in the stress response, or to only highly expressed genes (Brauer *et al*. 2008; Gasch *et al*. 2000) (Figure S6**)**. Importantly, the correlation between increased expression and predicted displacement from the nuclear periphery holds for both subtelomeric and nonsubtelomeric genes (Figure 7, C and D). Examples of correlated changes in expression and localization are shown for the *TEL4R*-proximal region, which is perinuclear in wild-type cells but is displaced away from the nuclear periphery in FC(IV:XII) (Figure 6F), and presumably in all other FC strains, as this region is always engaged in fusions (Figure 3). Most genes in this region show increased expression after predicted displacement toward the nuclear interior (Figure 7E). Of the almost 500 genes that were predicted to change their peripheral localization by >5%, 85 experienced changes in expression (Figure 7F, listed in Tables S3–S4).

### The effects of nuclear location *vs.* centromere and telomere deletion

**Figure 8:**
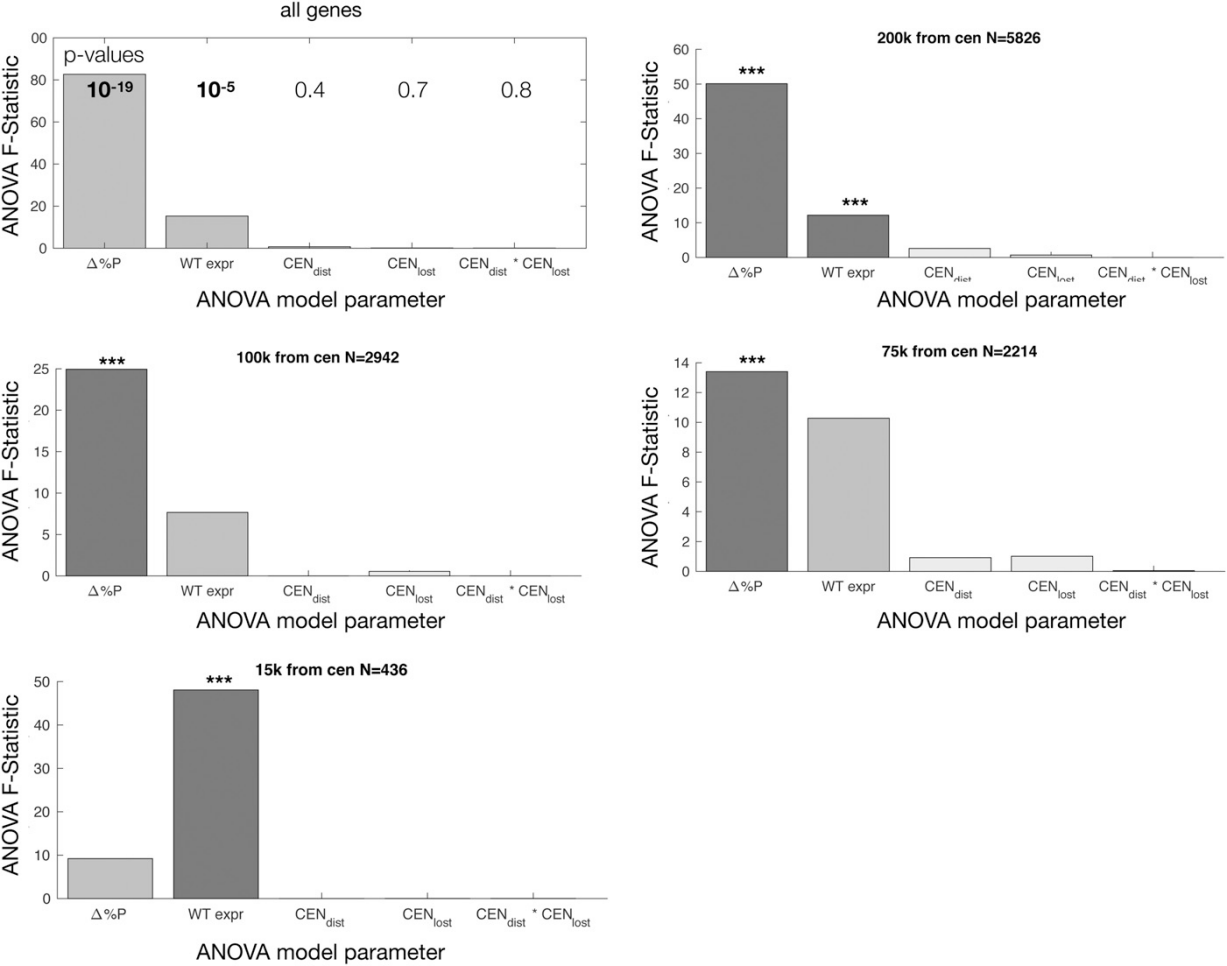
Deletion of the *CEN* element does not account for changes in the expression of nearby genes. ANOVA F-Statistic (the predictive power of each variable) for measured change in expression [log2(FC/WT)] for all genes, or only genes 200, 100, 75, or 15 kb from the centromere. Multivariate ANOVA shows that the only significant predictors of change in expression are the predicted change in localization relative to the nuclear periphery (Δ%P) and, to a lesser extent, the expression of that gene in WT cells (*** *P* < 0.05 after multiple hypothesis testing). In this ANOVA, terms are added sequentially, so the model is testing if WT_expr_ adds to the predictive power of a model that already includes Δ%P, then tests if adding CEN_dist_ further improves the model, and so on. CEN_dist_ tests if the distance to the centromere is correlated with changes in expression after taking into account all other features in the model (change in %peripheral, WT expression, etc). CEN_lost_ tests if deletion of a CEN element affects expression after taking into account all other features in the model (change in %peripheral, WT expression, etc). CEN_dist_*CEN_lost_ (the last bar) is an interaction term testing if the distance to the centromere specifically matters for deleted centromeres. Cen, centromere; expr, expression; FC, fused chromosome; WT, wild-type.

Increased expression could be caused by deletion of repressive elements in telomeres and centromeres during chromosome fusion, gene displacement away from the nuclear periphery, or from a combination of these two factors. As deletion of the centromere is highly correlated with changes in location, to determine if deletion of a centromere affects expression, we used ANOVA to determine if the distance to the centromere is predictive of changes in gene expression after first taking into account predicted changes in location and wild-type gene expression. We find that after taking into account the predicted change in location, the distance to the centromere (whether deleted or not) is not predictive of changes in expression (Figure 8). This suggests that spreading of a repressive signal in *cis* around centromeres is unlikely to measurably affect expression, and that distance to the nuclear periphery may be the dominant effect.

**Figure 9:**
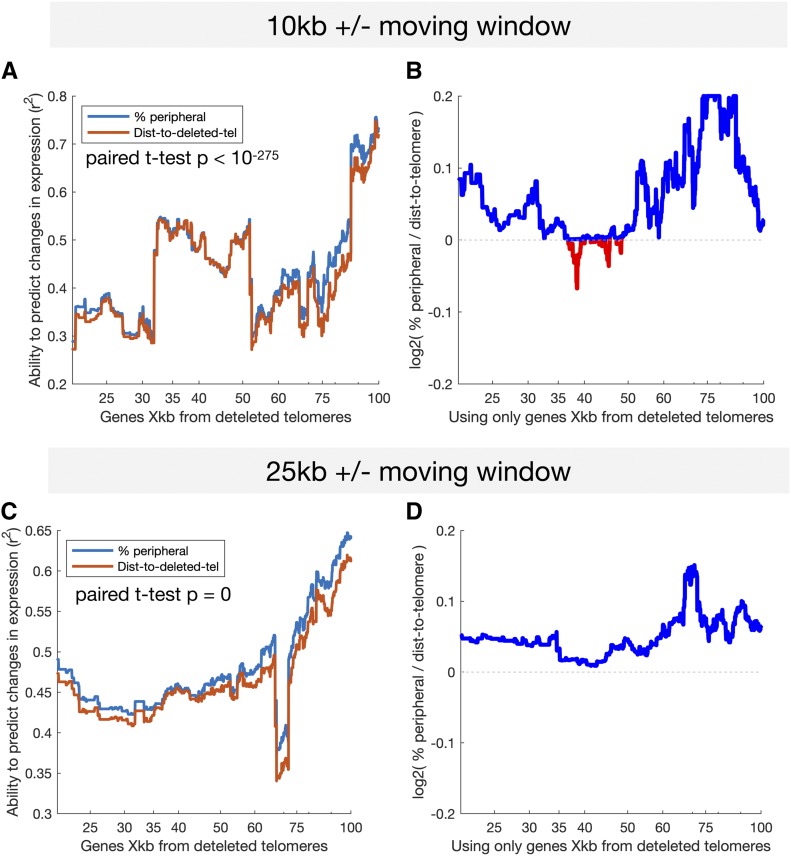
The predicted change in % peripheral is a better predictor of changes in gene expression than is the distance to the telomere. Using data from all FC strains, we selected the subset of genes on all chromosome arms that underwent fusion, and calculated the fold-change in expression (relative to wild-type), the change in % peripheral, and the distance to the former telomere. (A and C) The r^2^ for predicting change in expression as a function of either % peripheral or distance to the telomere for all genes within a 20- (A) or 50-kb (B) moving window. This measures the correlation of each feature within each window. (B and D) Each point shows the fold difference between the ability of changes in % peripheral to predict expression and the ability of the log distance to the telomere to predict expression. Each value is the log2(r^2^_%peripheral_/r^2^_dist-to-tel_) for the set of genes that are in a 20- (B) or 50-kb (D) moving window centered X kb from the former telomere. Gene sets in which changes in % peripheral are better predictors of changes in expression (log2(r^2^_%peripheral_/r^2^_dist-to-tel_) > 0) are colored blue. dist-to-tel, distance to telomere; FC, fused chromosome.

Deletion of telomeres may affect the expression of subtelomeric genes. To measure the effects of telomere deletion on gene expression, we used all FC strains and the subset of genes on a chromosome arm that underwent fusion and telomere loss, and predicted changes in expression from both distance to the deleted telomere and from the predicted frequency in the nuclear periphery. We then asked which is a better predictor of changes in gene expression. For the subset of genes on chromosome arms that underwent fusion, we took all genes X kb (+/− 10 kb) from the deleted telomere, and used a linear model to predict changes in expression from % peripheral or from distance to the deleted telomere for this set of genes, allowing us to correlate changes in expression with each feature. Thus, we obtained an r^2^ for each, and the feature with the higher r^2^ is the better predictor (Figure 9, A and C). Taking the log2(ratio) of the r^2^ values, if the log2(ratio) is >0, then % peripheral is a better predictor. While both features are similarly predictive, increased expression correlates better with predicted frequency in the nuclear periphery for genes that are both close to the deleted telomere, as well as for genes further away (Figure 9, B and D). This suggests that these expression changes are not, or not entirely, due to distance from the deleted telomere, and that distance from the periphery plays a slightly more important role.

## Discussion

Interphase yeast chromosomes are organized with centromeres clustering around the SPB, telomeres associating with the NE, and chromosome arms extending between these two anchoring points in a brush-like fashion. How this organization affects nuclear functions is not fully understood. Previous studies have reported altered expression of subtelomeric genes in mutants that disrupt heterochromatin formation or telomere clustering (Wyrick *et al.* 1999; Taddei *et al.* 2009). Importantly, these studies did not directly address the role of 3D chromosome organization, as the genetic perturbations used (depletion of histone H4, and mutations of the silencing factor *SIR3* and of the telomere tethering proteins *YKU70* and *ESC1*) affected multiple processes, including heterochromatin formation, genome-wide gene expression, and DNA repair.

In this study, we used tailored chromosome fusions (FC cells) to alter interphase nuclear organization in otherwise wild-type cells. Computational modeling validated with single-cell imaging revealed significant changes in nuclear organization after these chromosome fusion events. These changes included displacement of donor chromosomes away from the SPB after deletion of their centromeres and displacement of chromosome regions away from the nuclear periphery after deletion of neighboring telomeres. Furthermore, the distance between two chromosome loci in the arm of a donor chromosome was reduced upon fusion to a receiving chromosome, in both live cells and computational models. Notably, reduced distances between the same chromatin loci in FC strains were previously observed during anaphase chromosome segregation (Neurohr *et al.* 2011; Titos *et al.* 2014). This suggests that physical constraints acting on interphase chromatin of fused donor chromosomes can lead to their increased compaction, which is then maintained throughout the cell division cycle. This highlights the power of polymer-based modeling to reproduce nuclear organization features (Tjong *et al.* 2012; Wong *et al.* 2012) and further extends the applicability of these approaches to predict nuclear organization of yeast strains with chromosome fusions, based only on minimal imposed constraints.

Our analysis reveals that genome-wide gene expression levels remained generally unaffected by changes in chromosome organization. However, we also find that chromosome fusions result in consistent and reproducible increases in expression, with >100 genes exhibiting a mild but significant increase. This is consistent with normal growth of FC strains in rich media (Titos *et al.* 2014), and with recent reports that overall transcription is not affected by fusion of all yeast chromosomes into one or two mega-chromosomes (Luo *et al.* 2018; Shao *et al.* 2018). These two studies also reported derepression of subtelomeric genes near chromosome fusion sites, which was attributed to disruption of telomeric silencing. These studies used one to three RNA-seq biological replicates, whereas we used four biological replicates for wild-type, and three or four biological replicates for each of 10 FC genotypes, for a total of 42 experiments. Accurate quantification of expression changes of <50% requires >10 replicates (Schurch *et al.* 2016), potentially explaining why we identified a relatively higher number of genes with changes in expression of 10–20%. Because increased expression of these genes is correlated with both their 1D distance to the former telomere and their 3D distance to the periphery, both deletion of neighboring telomeres and spatial displacement away from the nuclear periphery may contribute to increased expression levels of subtelomeric genes. Our results suggest that, while deletion of telomere sequences may play a role, 3D distance to the periphery is likely a major factor affecting gene expression (Figure 9).

It is interesting to consider our results in the context of previous studies on the mechanisms of subtelomeric silencing in budding yeast. Transcription levels are known to decrease in proximity to telomeres [reviewed in Mondoux and Zakian (2006)]. Moreover, gene targeting to the nuclear periphery, either by integration of reporters in subtelomeric regions or by artificial anchoring to perinuclear proteins, leads to silencing that is dependent on perinuclear enrichment of SIR factors (Gottschling *et al.* 1990; Andrulis *et al.* 1998; Pryde and Louis 1999; Taddei *et al.* 2009). These observations led to the hypothesis that the NE is a transcriptionally repressive environment due to the local accumulation of repressive factors. However, a truncated telomere that does not localize to the nuclear periphery can still support silencing of a *URA3* reporter (Mondoux *et al.* 2007), and microarray analysis has shown that almost 80% of subtelomeric genes are still silenced after telomere detachment from the nuclear periphery in *esc1 yku70* mutants (Taddei *et al.* 2009). These findings raised the possibility that subtelomeric gene position and expression are independent of each other. In contrast, our results suggest that displacement from the nuclear periphery affects the expression levels of native subtelomeric genes, but that this effect is relatively mild, which may have escaped previous analysis using growth on selective media or microarrays. These findings support the hypothesis that regulation of perinuclear localization of subtelomeric genes (*e.g.*, by telomere detachment) may affect their expression in response to environmental signals. Since chromosome detachment in the FC strains examined here caused relatively mild changes in expression, it remains unclear to what extent changes in position may contribute to the induction of subtelomeric gene expression in stress conditions.
